# Inverse correlation of HER2 with MHC class I expression on oesophageal squamous cell carcinoma

**DOI:** 10.1038/sj.bjc.6605772

**Published:** 2010-07-13

**Authors:** T Maruyama, K Mimura, E Sato, M Watanabe, Y Mizukami, Y Kawaguchi, T Ando, H Kinouchi, H Fujii, K Kono

**Affiliations:** 1 First Department of Surgery, University of Yamanashi, 1110 Shimokato, Chuo-city, Yamanashi 409-3898, Japan; 2 Department of Neurosurgery, University of Yamanashi, 1110 Shimokato, Chuo-city, Yamanashi 409-3898, Japan; 3 Department of Orthopaedic Surgery, University of Yamanashi, 1110 Shimokato, Chuo-city, Yamanashi 409-3898, Japan

**Keywords:** MHC class I, oesophageal cancer, HER-2, CTL

## Abstract

**Background:**

As HER2 is expressed in 30% of oesophageal squamous cell carcinomas (ESCCs), T-cell-based immunotherapy and monoclonal antibodies targeted against HER2 are attractive, novel approaches for ESCCs. However, it was shown that there is an inverse correlation between HER2 and MHC class I expression on tumours. Thus, the correlation between HER2 and MHC class I expressions on ESCC was evaluated.

**Methods:**

Expressions of MHC class I and HER2 in ESCC tissues (*n*=80) and cell lines were assessed by immunohistochemistry, fluorescence *in situ* hybridisation (FISH), and flow cytometry. We investigated whether HER2 downregulation with small interfering RNA (siRNA) in ESCC cell lines could upregulate the expression of MHC class I and the antigen presentation machinery components, and could increase their sensitivity for tumour antigen-specific CTLs.

**Results:**

There was an inverse correlation between HER2 and MHC class I expressions in both tumour tissues and cell lines. Downregulation of HER2 with siRNA resulted in the upregulation of MHC class I expression, leading to increased CTL recognition by tumour antigen-specific CTLs.

**Conclusion:**

HER2-overexpressing ESCC tumour cells showed a reduced sensitivity for CTLs through the downregulation of MHC class I.

The HER2/neu-proto-oncogene (HER2) is a member of the epidermal growth-factor receptor family, and encodes a 185-kDa transmembrane glycoprotein with tyrosine-specific kinase activity (Coussens *et al*, 1985). HER2 is amplified and overexpressed in 15–20% of breast cancers (Slamon *et al*, 1989), and also in a variety of other tumours such as ovarian carcinomas (Slamon *et al*, 1989), colorectal cancers (Al-Kuraya *et al*, 2007), gastric cancers, and oesophageal squamous cell carcinomas (ESCCs) (Takehana *et al*, 2002; Mimura *et al*, 2005). Because of its selective overexpression in malignant tissue, HER2 has been used as a target for immunotherapy, including humanised monoclonal antibodies (Herceptin) and T-cell-based immunotherapy (Fisk *et al*, 1995; Disis and Cheever, 1997; Baselga *et al*, 1998; Kono *et al*, 1998, 2002; Murray *et al*, 2000). Recent articles have shown that collaboration between both humoural and cellular responses is required for the complete eradication of HER2-expressing tumours (Knutson *et al*, 2001; Reilly *et al*, 2001; Vertuani *et al*, 2009).

The prognosis of advanced ESCC patients remains poor despite the use of combined modality therapy (Kato *et al*, 1991; Akiyama *et al*, 1994; Hayashi *et al*, 2001; Ishikura *et al*, 2003). As HER2 was reportedly expressed in 30.3% of ESCC patients (Mimura *et al*, 2005), T-cell-based immunotherapy and monoclonal antibodies targeted against HER2 are therefore attractive approaches as a novel immunoadjuvant therapy for ESCC patients.

As tumour-reactive CTLs lyse tumour cells through the recognition of tumour antigens on MHC class I, the downregulation of MHC class I expression and antigen-processing machinery (APM) components are often associated with reduced sensitivity to lysis by CTLs (Goodenow *et al*, 1985; Ferrone and Marincola, 1995; Seliger *et al*, 1998). Reduced MHC class I expression on tumours is often associated with disease progression and a poor prognosis in ovarian, colorectal, and breast cancer (Zia *et al*, 2001; Watson *et al*, 2006; Leffers *et al*, 2008). Recently, we also reported that the downregulation of MHC class I molecules is associated with a poor prognosis in patients with ESCC (Mizukami *et al*, 2008). It is noteworthy that it was shown that HER2 expression is associated with an immune escape phenotype. For example, it was reported that there was an inverse correlation between HER2 and MHC class I expression in murine tumour models (Lollini *et al*, 1998; Herrmann *et al*, 2004; Kaplan *et al*, 2006); in addition, the inverse correlation between HER2 and TAP expression resulted in reduced sensitivity to lysis by CTLs in human tumours (Herrmann *et al*, 2004). Thus, there is a possibility that HER2-overexpressing tumours might escape from CTLs specific for tumour antigens, because of the downregulated MHC class I and APM components on the tumours. However, there is still limited information describing the relationship between HER2 and MHC class I expressions on human tumours.

The aims of this study were to analyse: (1) the correlation between HER2 and MHC class I expressions in ESCC; (2) the relationship between the inhibition of HER2 signalling and expressions of MHC class I and APM components in ESCC; and (3) CTL function as a consequence of HER2 signal inhibition in ESCC.

## Materials and methods

### Reagents

The following reagents were purchased from Sigma-Aldrich (St Louis, MO, USA): Tween-20, penicillin streptomycin, human serum albumin, and dimethyl sulfoxide (DMSO). Phycoerythrin (PE)-conjugated mouse anti-HER2/neu antibody (Becton Dickinson, Franklin Lakes, NJ, USA), PE-conjugated mouse IgG1 *κ* isotype control immunoglobulin (Becton Dickinson), PE-conjugated mouse anti-human HLA-A2 (Becton Dickinson), PE-conjugated mouse IgG2b *κ* isotype control (Becton Dickinson), FITC-conjugated mouse anti-human HLA-ABC (W6/32; eBioscience, San Diego, CA, USA), and FITC-conjugated mouse IgG2a, *κ* isotype control (eBioscience) were used for flow cytometric analysis. Rabbit anti-HER2/ErbB2 antibody (Cell Signalling Technology, Danvers, MA, USA), rabbit anti-phosphorylated HER2/ErbB2 (Tyr1221/1222) antibody (Cell Signalling Technology), rabbit anti-LMP2 antibody (Affinity, Mamhead, UK), rabbit anti-LMP7 antibody (Affinity), rabbit anti-TAP1 antibody (StressGen, Victoria, Canada), rabbit anti-Tapasin antibody (StressGen), and rabbit anti-Actin antibody (Cell Signalling Technology) were used as primary antibodies for western blot analysis.

### Cell lines

Oesophageal squamous cell carcinoma cell lines TE-1, TE-3, and TE-4 were a kind gift from Dr Nishihara (Institute of Development, Aging and Cancer, University of Tohoku, Sendai, Japan). ESCC cell lines KYSE-30 and KYSE-50 were purchased from the Health Science Research Resources Bank (Osaka, Japan). ESTDAB049 (melanoma cell line) and HTB122 (breast cancer cell line) were a kind gift from Dr Rolf Kiessling (Karolinska Hospital, Stockholm, Sweden). KATO III (gastric cancer cell line) and PC-9 (lung cancer cell line) were obtained from the IBL cell bank (Gunma, Japan). SK-BR-3 (breast cancer cell line) and BT474 (breast cancer cell line) were obtained from American Type Culture Collection (Manassas, VA, USA). The TISI cell line comprises HLA-A24+, Tap– cells derived from a human B-lymphoblastoid cell line. These cell lines were kept in RPMI-1640 (Invitrogen, Carlsbad, CA, USA) with 5% FCS (Invitrogen), 50 U ml^–1^ penicillin, and 2 mM L-glutamine.

### Patients and samples

A total of 80 consecutive patients with primary ESCCs who were histologically diagnosed and treated in the First Department of Surgery, University of Yamanashi Hospital, were enrolled in this study. None of the patients had received any treatment before surgery (preoperative radiotherapy, chemotherapy, or immunotherapy) and all patients had undergone oesophagectomy with two- or three-field lymph node dissection. This study was approved by the ethical committee of the University of Yamanashi, and written informed consent was obtained from all individuals.

### Immunohistochemical (IHC) analysis

Sections of archival, formalin-fixed, and paraffin-embedded material, 4-*μ*m thick, were used for IHC analysis. For HER2 staining, HercepTest (DaKoCytomation, Glostrup, Denmark) was performed according to the manufacturer's recommendations. In brief, deparaffinized and rehydrated sections were incubated with Epitope Retrieval Solution (DaKoCytomation) for 40 min at 99°C. The sections were washed and blocked with 3% hydrogen peroxide for 5 min. The sections were then washed and incubated with the primary antibody, a rabbit polyclonal antibody to human HER2, or the primary negative control antibody at room temperature for 30 min. After washing, the primary antibody was detected using visualisation reagents for 30 min of incubation at room temperature. Subsequently, diaminobenzidine was applied as a visualisation reagent for 10 min and the section was counterstained with haematoxylin. Positive and negative control slides provided with the HercepTest were used in this study. Two observers (YM and KM) performed the IHC analysis according to the manufacturer's criteria, without previous knowledge of the clinicopathological data.

For MHC class I staining, the sections were dewaxed, followed by antigen retrieval with Epitope Retrieval Solution (DakoCytomation) in an autoclave for 20 min at 121°C. The sections were blocked by Chemmate Peroxidase Blocking Solution (DakoCytomation). The primary antibody EMR8-5 (Cosmo Bio Co., Tokyo, Japan) or the isotype control monoclonal antibody (DakoCytomation) was applied to the sections at 4°C overnight. The sections were incubated with streptavidin–biotin complex (Nichirei, Tokyo, Japan) for 30 min, and then they were treated with 3,3′-diaminobenzidine (DakoCytomation) for 5 min, and counterstained with haematoxylin. The staining intensity was evaluated using the following criteria: strong positive (strong), dark brown staining in >50% of tumour cells completely obscuring the cytoplasm; and weak positive (weak), any lesser degree of brown staining appreciable or no appreciable staining in tumour cells. Two observers (YM and TM) performed the IHC analysis without previous knowledge of the clinicopathological data.

### FISH analysis

Fluorescence *in situ* hybridisation (FISH) analysis was performed using the PathVysion HER2 DNA Probe Kit (Abbott Molecular, Abbott Park, IL, USA). The HER2/neu-SpectrumOrange probe is specific for the *HER2* gene locus, and the CEP 17-SpectrumGreen probe is specific for the *α*-satellite DNA sequence. The FISH procedures were performed according to the manufacturer's protocol. In brief, the sections were deparaffinized, dehydrated, and incubated in 20% sodium bisulfate/2 × standard saline citrate at 43°C for 20 min. The sections were washed and treated with proteinase K (Boehringer-Mannheim, Mannheim, Germany) at 37°C for 25 min. Subsequently, denaturation, hybridisation, and post-hybridisation washing were performed according to the manufacturer's protocol. After hybridisation and post-hybridisation washing, the sections were counterstained with DAPI (4′,6-diamidine-2′-phenylindole dihydrochloride). Using a fluorescence microscope (Olympus, Tokyo, Japan) equipped with Triple Bandpass Filter sets (Vysis, Downers Grove, IL, USA), FISH analysis was conducted. Signals were counted for at least 40 cancer nuclei per tumour and analysis was performed by two observers (KM and TM) according to the criteria provided with the PathVysion HER2 DNA Probe Kit. A breast tumour sample, which was previously identified as showing HER2 amplification and overexpression, was used as a positive control for HER2 FISH.

### Small interfering RNA (siRNA) transfection

The cell lines, TE1, TE3, TE4, and KYSE30, were grown in six-well plates until they reached approximately 60% confluence, and cells were then transfected with Opti-MEN I reduced serum medium (Invitrogen) including 100 pmol of siRNA (ERBB2 ON-TARGETplus SMARTpool siRNA or ON-TARGETplus siCONTROL non-targeting siRNA; Dharmacon, Inc., Chicago, IL, USA) or without siRNA using 5 *μ*l of Lipofectamine 2000 (Invitrogen) according to the manufacturer's protocol. After 36 h, cells were analysed by flow cytometry for HER2, MHC class I, and HLA-A2 expressions. These transfectants were always cultured in parallel for the same period of time. Three identical but independently derived transfectants were produced from TE1, TE3, TE4, and KYSE30 cell lines, with each set showing comparable results.

### HLA-A2402-restricted, cancer–testis antigen (CTA)-specific CTL generation

Peripheral blood mononuclear cells (PBMCs) were collected from a healthy HLA-A2402-positive volunteer using Ficoll Paque (GE Health Care, Uppsala, Sweden) gradient centrifugation. Monocytes were enriched by adherence to a plastic tissue culture flask for 90 min at 37°C. Adherent cells were cultured in X-Vivo (Life Technologies Inc., Gaithersburg, MD, USA) supplemented with 1000 units ml^–1^ of GM-CSF (Peprotech EC Ltd, London, UK) and 1000 units ml^–1^ of IL-4 (Peprotech EC Ltd). On day 5, immature DCs were treated with 20 *μ*g ml^–1^ of OK-432 (Chugai Pharmaceutical Co., Tokyo, Japan) and, after 48 h, these treated cells were used as mature DCs. On day 7, mature DCs were pulsed with 20 *μ*g ml^–1^ of HLA-A2402-restricted URLC10 peptide (RYCNLEGPPI), which is immunodominant peptide derived from CTA specific for ESCC (Suda *et al*, 2007), for 1 h at 37 °C and subsequently co-incubated with autologous PBMCs in a 1 : 10 ratio in a 24-well plate. X-Vivo medium was used as the culture medium and supplemented with 20 IU ml^–1^ interleukin-2 (IL)-2; Shionogi & Co., Ltd, Osaka, Japan) after 2 days of culture. Cultured cells were re-stimulated with peptide (URLC-10)-loaded, irradiated (60 Gy) autologous PBMCs on days 14 and 21. A total of 50–100 IU ml^–1^ of IL-2 was replenished 2 days after each re-stimulation. At 1 week after the second re-stimulation, CTLs were tested for their antigen-specific response using the ELISpot assay based on the combination of HLA-A2402 binding, irrelevant peptide, TTK (SYRNEIAYL), and KOC-1 (KTVNELQNL).

### Western blotting

Cell pellets were solubilised in electrophoresis sample buffer, and sonicated for 10 s, and then boiled for 10 min. The protein concentration of cell lysates was measured, and the same amount of protein (30 *μ*g) was separated by SDS–PAGE, followed by transfer to a polyvinylidene fluoride (PVDF) microporous membrane (Millipore, Billerica, MA, USA) blocked in PBS (5% milk). After blocking, the membrane was probed with primary antibodies. The membranes were again washed and incubated with HRP-linked goat anti-rabbit antibody (Cell Signalling Technology) or alkaline phosphatase-conjugated swine anti-rabbit antibody (DakoCytomation). The specific bands were visualised by ECL Plus (Amersham Pharmacia Biotech AB, Uppsala, Sweden) or CDP-Star substrate (Roche, Mannheim, Germany), and images were digitally captured using a LAS1000 Lumino Image Analyser (Fuji Photo Film, Tokyo, Japan).

### ELISpot assay

The ELISpot assay was performed using a commercial kit (Mabtech, Stockholm, Sweden), according to the manufacturer's protocol. In brief, 96-well plates with nitrocellulose membranes (Millipore) were coated with a primary anti-human IFN-*γ* capture monoclonal antibody (1-D1K) overnight. The plates were then treated with X-Vivo containing 1% human serum albumin for 90 min. Target cells (2 × 10^4^ per well) and CTLs (2 × 10^3^ per well) were co-incubated in each well with 200 *μ*l of X-Vivo for 24 h. Thereafter, a biotinylated secondary anti-human-IFN-*γ* monoclonal antibody (7-B6-1) was added for 2 h and streptavidin–alkaline phosphatase reagent was added for 1 h, followed by staining with NBT and BCIP (Invitrogen). The number of spots was quantified using an auto-analysing system, KS ELISPOT Compact (Zeiss, Göttingen, Germany).

### IFN-*γ* treatment of ESCC

Oesophageal squamous cell carcinomas (TE4, TE1, TE3, KYSE30, and KYSE50) were incubated with or without IFN-*γ* (10 ng ml^–1^; R&D Systems, Minneapolis, MN, USA) for 24 h in X-Vivo medium. Thereafter, MHC class I and HER-2 expressions were analysed by flow cytometry.

### Flow cytometry

Cell staining was performed according to standard flow cytometric staining protocols, and samples were analysed using a four-colour FACS machine (FACSCalibur, Becton Dickinson).

### DNA typing of HLA in tumour cell lines

DNA typing of HLA-A gene polymorphisms was performed by the polymerase chain reaction–sequencing-based typing (PCR-SBT) method (Reeve and Fuller, 1995).

### Statistics

The *χ*^2^ test was applied to examine the differences between HER-2 and MHC class I expressions in ESCC. To evaluate statistical differences between the two groups, the non-paired Student's *t*-test was used. Significance was accepted at *P*-values of <0.05.

## Results

### Expressions of HER2 and MHC class I on ESCC tissues analysed by immunohistochemistry and FISH

**Figure 1:**
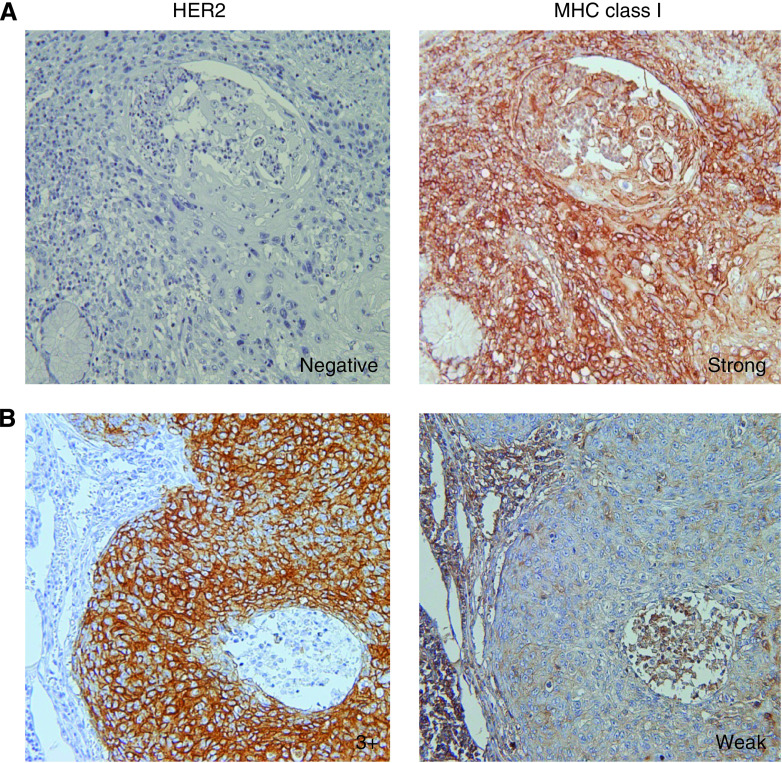
Representative MHC class I immunohistochemical analysis of HercepTest 0 (**A**) and 3+ (**B**) cases. The serial sections were analysed using HercepTest and MHC class I staining. (**A**) MHC class I staining was strongly positive in a HercepTest 0 case. (**B**) MHC class I staining was weakly positive in a HercepTest 3+ case. Original magnification × 200.

A total of 80 consecutive patients with primary ESCCs were analysed by immunohistochemistry for HER2 and MHC class I expressions, and by FISH for *HER2* gene amplification. Representative IHC stainings of HER2 and MHC class I (EMR8-5 mAb) on serial sections are shown in Figure 1. Although HER2-negative case showed strong positive staining of MHC class I (Figure 1A), strong HER2-positive (HercepTest 3+) cases showed weak positive staining of MHC class I (Figure 1B). Summarised data from the 80 cases indicated that there was an inverse correlation of *HER2* gene amplification with MHC class I expression (Table 1, *P*=0.002 by *χ*^2^ analysis). These observations suggested that HER2-positive ESCC showed downregulated MHC class I, whereas HER2-negative ESCC showed preserved MHC class I.

### HER2 and MHC class I expressions on tumour cell lines

Next, HER2 and MHC class I expressions (W6/32 mAb) were analysed by flow cytometry using a panel of 11 tumour cell lines including ESCC cell lines. Tumour cell lines were divided into HER2 low- and HER2 high-expression groups according to the levels of HER2 (Table 2). All HER2-high cell lines, TE4, SKBr3, and BT474, showed HercepTest 3+ by immunocytochemistry and *HER2* gene amplification by FISH (data not shown). As a result, MHC class I expression in the HER2 high-expression group was significantly downregulated in comparison to those in the HER2 low-expression group (34.8±17.1 *vs* 221.4±116.6, *P*=0.0175). These results further support the inverse correlation between HER2 and MHC class I expression.

### HER2 siRNA transfection enhanced MHC class I expression

To further evaluate the relation of HER2 expression with MHC class I expression, we performed siRNA techniques targeting HER2 to inhibit HER2 signalling and expression. Four ESCC cell lines (TE1, TE3, TE4, and KYSE30) were transfected with siRNA targeting HER2 and assessed by flow cytometric analysis and western blot.

**Figure 2:**
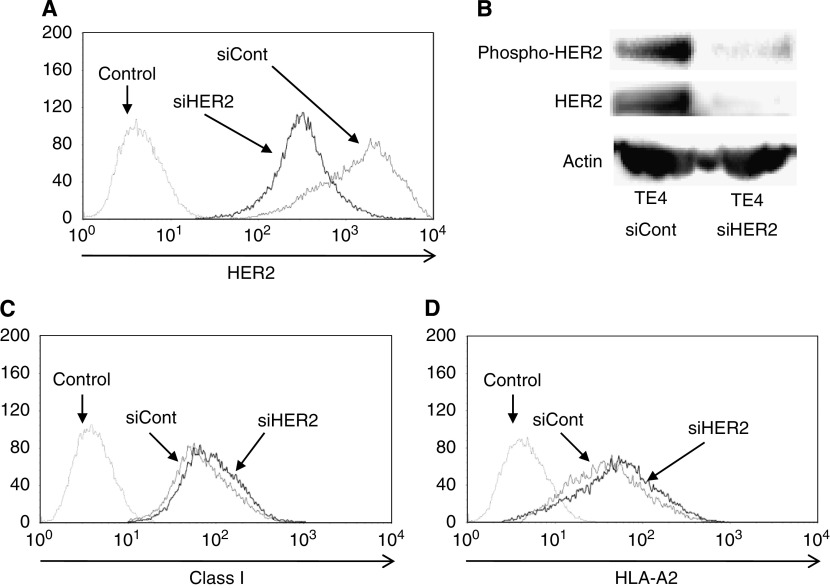
MHC class I expression after HER2 downregulation by siRNA targeting HER2. TE4 (ESCC cell line) was transfected with siRNA targeting HER2 (siHER2) or the control (siCont). (**A**) HER2 expression was downregulated in siHER2 transfectants, as assessed by flow cytometric analysis. (**B**) Total and phosphorylated HER2 expressions were downregulated in siHER2 transfectants, as assessed by western blot. MHC class I expression (**C**) and HLA-A2 (**D**) were increased in siHER2 transfectants, as assessed by flow cytometric analysis. The data were confirmed in four independent experiments, and representative data are shown.

As shown in Figure 2A and B, treatment of TE4 with HER2-siRNA transfection resulted in the downregulation of total HER2 and phosphorylated HER2 protein in comparison to those in control siRNA transfectants. It is noteworthy that total MHC class I and HLA-A2 expressions in TE4-HER2-siRNA transfectants were upregulated in comparison to those in control siRNA transfectants (Figure 2C and D). Furthermore, even if ESCC cell lines (TE1, TE3, and KYSE30) were HER2 low-expressing cells (FISH negative), HER2 downregulation induced by HER2-siRNA transfection could upregulate total MHC class I and HLA-A2 expressions (Table 3). These observations were confirmed in four independent experiments. Taken together, the downregulation of HER2 induced by HER-2-siRNA transfection resulted in the upregulation of MHC class I expression.

**Figure 3:**
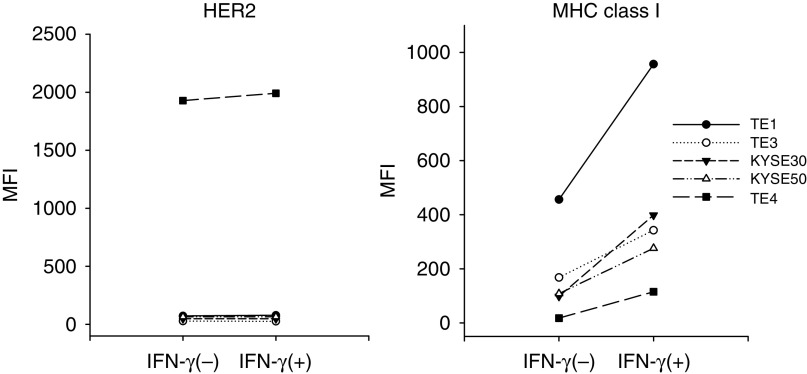
IFN-*γ* treatment of ESCC cell lines. ESCCs (TE4, TE1, TE3, KYSE30, and KYSE50) are incubated with or without IFN-*γ* (10 ng ml^–1^) for 24 h in X-Vivo medium. Thereafter, MHC class I and HER-2 expressions are analysed by flow cytometry.

We next investigated whether MHC class I expression on ESCCs (TE4, TE1, TE3, KYSE30, and KYSE50) is upregulated after IFN-*γ* treatment. As show in Figure 3, IFN-*γ* treatment resulted in the upregulation of MHC class I expression on all of the ESCCs tested, whereas the HER-2 expression on ESCC did not alter after IFN-*γ* treatment.

### Downregulation of HER2 enhanced HLA-A2402-restricted URLC10-specific CTL recognition

**Figure 4:**
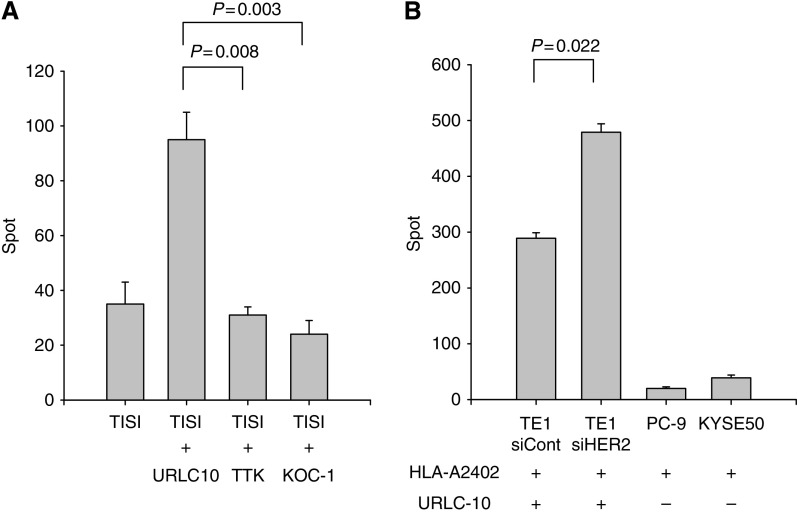
HER2 downregulation and CTL recognition. (**A**) URLC10 is an HLA-A2402-restricted peptide derived from cancer–testis antigen specific for ESCC. The URLC10-specific CTL line was generated from an HLA-A2402 (+) healthy donor and tested for specificity against TISI targets pulsed with cognate peptide (URLC10) or irrelevant HLA-A2402-restricted peptides (TTK and KOC-1) using the ELISpot assay. (**B**) TE1 expressing URLC10 and HLA-A2402 was treated with siRNA targeting HER2 (siHER2) or the control (siCont) and subjected to the ELISpot assay with a URLC10-specific CTL line in combination with control tumour cell lines (PC-9 and KYSE50).

Subsequently, we investigated whether the HLA-A2402-restricted, tumour-specific antigen presentation on ESCC would be affected by the alteration of HER2 expression. We generated an HLA-A2402-restricted, URLC10-specific CTL line from a healthy HLA-A2402-positive volunteer, in which URLC10 is an immunodominant peptide derived from CTA specific for ESCC (Suda *et al*, 2007). The CTL line specific for URLC10 significantly recognised TISI pulsed with URLC10 peptide, as measured using the ELISpot assay, in comparison with TISI pulsed with irrelevant HLA-A2402 peptides (TTK and KOC-1, Figure 4A). It is noteworthy that the recognition of HER2-siRNA-treated TE1 by URLC10-specific CTLs was significantly enhanced in comparison to that for control siRNA-treated TE1 (Figure 4B). These results indicate that the downregulation of HER2 on ESCC resulted in upregulation of the tumour antigen-specific CTL response.

### Correlation of APM with HER2

**Figure 5:**
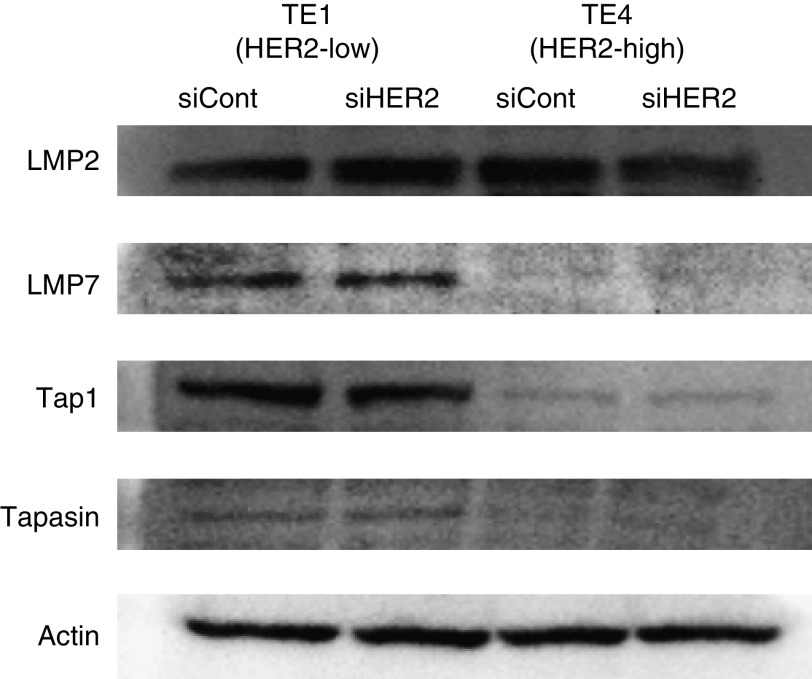
The expression of antigen-processing machinery (APM) components after HER2 downregulation. The expressions of APM components (LMP2, LMP7, Tap1, and tapasin) were assessed by western blot analysis against TE1 (HER2 low) and TE4 (HER2 high) transfected with siRNA targeting HER2 (siHER2) or the control (siCont).

We next further assessed APM components in relation to HER2 expression. ESCCs (TE1 and TE4) treated with HER2-siRNA and control siRNA were analysed by western blotting for APM components, including IFN*γ-*inducible immunoproteasome subunit low-molecular-mass polypeptide (LMP)-2, LMP-7, the transporter associated with antigen processing (TAP)-1, and tapasin, a member of the peptide loading complex. As a result, the expressions of TAP-1, LMP-7, and tapasin were downregulated or lost in HER2 high-expressing TE4 in comparison to HER2 low-expressing TE1 (Figure 5). However, there were no significant differences in the expression of LMP-2, LMP-7, TAP-1, or tapasin between siHER2 and control transfectants in both cell lines (Figure 5).

## Discussion

This study provided novel and important findings relevant to the relationship between HER2 and MHC class I expressions on ESCCs. First, there was an inverse correlation between HER2 and MHC class I expressions in both tumour tissues and cell lines. Second, the downregulation of HER2 with siRNA transfection on ESCC cell lines resulted in the upregulation of MHC class I expression, leading to increased CTL recognition by tumour antigen-specific CTLs.

HER2 was considered an attractive target for immunotherapy because of its selective overexpression in several types of malignant tumours, including ESCCs (Fisk *et al*, 1995; Disis and Cheever, 1997; Baselga *et al*, 1998; Kono *et al*, 1998, 2002; Murray *et al*, 2000). However, it was also shown that HER2 expression is associated with an immune escape phenotype. For example, it was reported that there was an inverse correlation between HER2 and MHC class I expressions in murine tumour models (Lollini *et al*, 1998; Herrmann *et al*, 2004; Kaplan *et al*, 2006); in addition, the inverse correlation between HER2 and TAP expressions resulted in a reduced sensitivity to lysis by CTLs in human tumours (Herrmann *et al*, 2004). Furthermore, we previously showed that Herceptin, which inhibits HER2 signalling and downregulates HER2 expression on the cell surface, enhances MHC class I-restricted antigen presentation recognised by HER2-specific CTLs (Kono *et al*, 2004). Thus, there is a possibility that HER2-overexpressing tumours might be able to escape from CTLs specific for tumour antigens, because of the downregulated MHC class I on the tumour. In this study, this phenomenon was further confirmed in human ESCC, in which there was an inverse correlation of HER2 expression with MHC class I expression. Furthermore, the downregulation of HER2 with siRNA transfection on ESCC cell lines resulted in the upregulation of MHC class I expression, leading to increased CTL sensitivity.

In this study, the phenomenon that downregulation of HER2 with siRNA transfection results in the upregulation of MHC class I expression occurred more markedly in HER2-overexpressing than in HER2 low-expressing ESCCs. It was also reported that the effects of HER2 expression on MHC class I-restricted antigen presentation are not universal among tumours with different levels of HER2 expression (Disis *et al*, 2004). Thus, the reason why HER2-expressing tumours do not always show a reduced MHC class I expression could be the requirement for a critical ‘threshold’ of HER2 expression.

Herrmann *et al* (2004) showed an inverse correlation between HER2 and TAP expressions in a human model. In the present study, although the expressions of TAP-1, LMP-7, and tapasin were downregulated or lost in HER2 high-expressing TE4 in comparison to HER2 low-expressing TE1, we could not detect significant alterations in the expressions of LMP2, LMP7, Tap1, and tapasin between ESCC treated with HER2-siRNA and the control. This discrepancy may be because of the limitation of the western blot assay system to detect the difference in APM components. Alternatively, other unknown APM components may be involved in the downregulation of MHC class I on ESCC.

The underlying mechanisms behind the downregulation of HER2 that could induce upregulation of MHC class I are currently unknown. It has been shown that oncogenes, such as ras and myc, can induce the downregulation of MHC class I surface expression, resulting in an escape from immunosurveillance (Basolo *et al*, 1992; van ‘t Veer *et al*, 1993; La Cava *et al*, 1994; Griffioen *et al*, 1995; Cheng *et al*, 1996). Furthermore, it has been shown that HER2 can activate several signal transduction pathways, including the ras/mitogen-activated protein kinase (MAPK) and PI3K/Akt pathways (Marmor *et al*, 2004). Recently, it has been shown that HER2-inhibition resulted in the upregulation of MAPK pathway in breast cancer cells (Lucci *et al*, 2010). Furthermore, Sers *et al* (2009) showed that interference with MAPK activation could upregulate HLA class I expression in colon cancer cells. These observations strongly suggested that alteration of the signal transduction cascades relating to HER2 may be involved in the expression of MHC class I and APM component. Taken together, the downregulation of HER2 with siRNA transfection on ESCC in this study might alter the signal transduction cascade, including MAPK or PI3K, and result in the upregulation of MHC class I expression.

In conclusion, regardless of the underlying mechanism of the HER2-induced downregulation of MHC class I, HER2-overexpressing ESCC tumour cells showed a reduced sensitivity for tumour antigen-specific CTLs, because of downregulation of MHC class I on their cell surface. These results suggest the possibility that alteration of the HER2-related signal with Herceptin, Lapatinib, or Rapamycin may regulate MHC class I expression on ESCC cells, leading to increased CTL sensitivity.

## Figures and Tables

**Table 1:** Correlation between HER2 and MHC class I in oesophageal squamous cell carcinoma

	**MHC class I expression**	
	**Weak**	**Strong**
FISH positive	7	1
FISH negative	24	48

Abbreviations: FISH=fluorescence *in situ* hybridisation; HER2=human epidermal growth factor receptor 2; MHC=major histocompatibility complex. The *χ*^2^ test, *P*=0.002 (*n*=80).

**Table 2:** HER2 and MHC class I expressions in tumour cell lines

	**HER2 (MFI)**	**Class I (MFI)**
TE4 (ESCC cell line)	687.0	43.4
SK-BR-3 (breast cancer cell line)	1570.2	49.4
BT474 (breast cancer cell line)	557.6	16.0
		
TE1 (ESCC cell line)	78.0	137.3
TE3 (ESCC cell line)	28.2	312.5
KYSE30 (ESCC cell line)	67.7	98.3
KYSE50 (ESCC cell line)	65.8	67.0
ESTDAB049 (melanoma cell line)	55.7	202.4
HTB122 (breast cancer cell line)	18.2	214.0
PC-9 (lung cancer cell line)	52.0	210.1
KATOIII (gastric cancer cell line)	114.8	313.8

Abbreviations: ESCC=oesophageal squamous cell carcinoma; HER2=human epidermal growth factor receptor 2; MFI=mean fluorescence intensity; MHC=major histocompatibility complex.

**Table 3:** HER2-siRNA effect on MHC class I and HLA-A2 expressions

			**HER2 (MFI)**		**MHC class I (MFI)**		**HLA-A2 (MFI)**	
**Cell line**	**HLA-A allele genotype**		**siCTR**	**siHER2**	**siCTR**	**siHER2**	**siCTR**	**siHER2**
TE1	2402	2601	67.6	20.6	118.8	158.1	Neg	Neg
TE3	0206	0206	33.3	11.7	245.8	290.3	246.7	269.7
TE4	0207	1101	605.2	304.5	40.4	73.4	33.6	76.6
KYSE30	0206	2402	78.5	26.3	115.4	165.9	93.8	148.8

Abbreviations: HER2=human epidermal growth factor receptor 2; MFI=mean fluorescence intensity; MHC=major histocompatibility complex; siCTR=control siRNA; siHER2=HER2 siRNA; neg=negative.
